# Millennium-Scale Crossdating and Inter-Annual Climate Sensitivities of Standing California Redwoods

**DOI:** 10.1371/journal.pone.0102545

**Published:** 2014-07-16

**Authors:** Allyson L. Carroll, Stephen C. Sillett, Russell D. Kramer

**Affiliations:** Department of Forestry and Wildland Resources, Humboldt State University, Arcata, California, United States of America; Montana State University, United States of America

## Abstract

Extremely decay-resistant wood and fire-resistant bark allow California’s redwoods to accumulate millennia of annual growth rings that can be useful in biological research. Whereas tree rings of *Sequoiadendron giganteum* (SEGI) helped formalize the study of dendrochronology and the principle of crossdating, those of *Sequoia sempervirens* (SESE) have proven much more difficult to decipher, greatly limiting dendroclimatic and other investigations of this species. We overcame these problems by climbing standing trees and coring trunks at multiple heights in 14 old-growth forest locations across California. Overall, we sampled 1,466 series with 483,712 annual rings from 120 trees and were able to crossdate 83% of SESE compared to 99% of SEGI rings. Standard and residual tree-ring chronologies spanning up to 1,685 years for SESE and 1,538 years for SEGI were created for each location to evaluate crossdating and to examine correlations between annual growth and climate. We used monthly values of temperature, precipitation, and drought severity as well as summer cloudiness to quantify potential drivers of inter-annual growth variation over century-long time series at each location. SESE chronologies exhibited a latitudinal gradient of climate sensitivities, contrasting cooler northern rainforests and warmer, drier southern forests. Radial growth increased with decreasing summer cloudiness in northern rainforests and a central SESE location. The strongest dendroclimatic relationship occurred in our southernmost SESE location, where radial growth correlated negatively with dry summer conditions and exhibited responses to historic fires. SEGI chronologies showed negative correlations with June temperature and positive correlations with previous October precipitation. More work is needed to understand quantitative relationships between SEGI radial growth and moisture availability, particularly snowmelt. Tree-ring chronologies developed here for both redwood species have numerous scientific applications, including determination of tree ages, accurate dating of fire-return intervals, archaeology, analyses of stable isotopes, long-term climate reconstructions, and quantifying rates of carbon sequestration.

## Introduction

**Figure 1:**
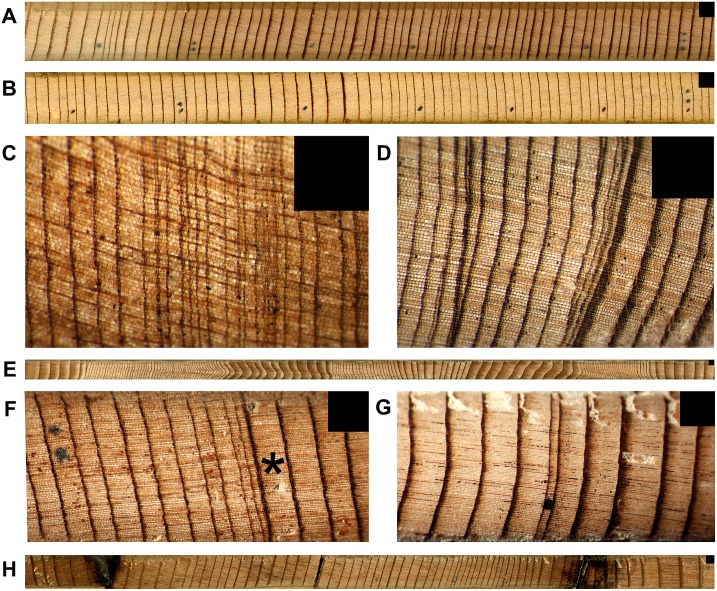
Tree-ring characteristics of *Sequoia sempervirens* and *Sequoiadendron giganteum*. (A) Crossdated SESE annual rings with marks beginning 1900 (three dots far right) and ending 1960 (one dot far left). (B) Crossdated SEGI annual rings with same years and marks as panel A. (C) Area of tight SESE annual rings, including missing rings. (D) SESE ring wedging, where discontinuous rings merge. (E) SESE spiral compression wood. (F) SESE annual rings from northernmost location (JS) showing 1739 event. After large 1738 ring (*), 1739 and 1740 are 1-cell-wide micro-rings merged with 1738 latewood, 1741 and 1742 rings are tight, and 1750 ring is marked with two dots on left. (G) SEGI annual rings from GF showing 1580 event, marked with one dot. Of 12 cores collected from the largest SEGI sampled, this is the only core showing 1580. (H) Two fire scars on core collected 45 m above ground in SESE rainforest (JS). Black boxes in upper right of each panel cover 1 mm^2^.

Coast redwood (*Sequoia sempervirens*, SESE) and giant sequoia (*Sequoiadendron giganteum*, SEGI) drew interest from early dendrochronologists because their decay-resistant heartwood, fire-resistant bark, and consequently great longevity provided access to intact millennium-scale tree-ring records. These are Earth’s tallest, heaviest, and among the oldest trees [1], [2]. Accurately dated tree-ring series of these species (Figure 1A and 1B) can thus provide a reliable basis for numerous scientific applications, including climate reconstructions and physiological analyses. While SEGI has been a cornerstone of modern dendrochronology, crossdating SESE has proven much more difficult. Accordingly, fundamental aspects of SESE dendrochronology, such as the development of range-wide tree-ring chronologies and assessment of potential drivers of year-to-year ring-width variation, have remained elusive.

During the early 1900s, A.E. Douglass formalized the precise dating of growth rings based on the common patterns of ring widths across a population of trees. The subsequent acceptance of crossdating as a valuable technique was due in part to Douglass’s success in developing a 3,200-year tree-ring chronology for SEGI [3]. Generally complacent growth rings (i.e., low degree of inter-annual variation) punctuated with strong marker years (i.e., consistently small or otherwise distinct rings) allowed accurate crossdating of this species [4]. SEGI chronologies have since been used to produce millennium-scale histories of fire [5] and drought [6]. Shortly after his pioneering work with SEGI, Douglass documented complicated and unsuccessful attempts to crossdate SESE [7].

Certain growth characteristics render SESE problematic for crossdating. Frequent discontinuous or missing annual rings (Figure 1C) reflect an absence of wood production at a given cambium location and preclude using simple ring counts to estimate tree age. Missing rings are often associated with a wedging or pinching pattern where multiple rings merge (Figure 1D) [8], [9]. Other confounding SESE growth attributes include patterns of spiral compression wood (Figure 1E) [9] and complacent rings with little annual variability [10]. Moreover, annual wood production occurs across an enlarging surface of cambium with increasing age, leading to narrower rings [11] even though whole-trunk wood volume growth continues to increase through old age [12].

Despite these challenges, there has been some progress crossdating SESE. Working on Douglass’ range-wide collection, Schulman crossdated sections from limited areas noting that while many samples showed non-climatic growth irregularities, some had “intelligible record[s]” of climate-limited annual rings, especially those from higher on trunks of co-dominant trees [13]. Indeed, the probability of obtaining a maximum ring count along one radius increased with height on main trunks of SESE in a second-growth forest and was higher for co-dominant versus suppressed trees [14]. A crossdated SESE chronology (1750–1985) created from partial cross-sections of 15 old-growth stumps and logs (>6 m above ground) was used to reconstruct fire history at Prairie Creek Redwoods State Park [10]. Unsuccessful attempts to crossdate SESE continued to be reported at sites across the range (e.g., Pt. Reyes National Seashore and Jackson State Demonstration Forest) as work on fire frequencies progressed [15], [16].

Climatic conditions generally drive the inter-annual variation in growth rings that underlies crossdating. Spatial synchrony of ring-width variation among sites and species points to broader-scale climate signals. Rather than closely tracking temperature or precipitation, SEGI’s rings better record extreme events such as severe drought [6], [17], [18]. There is minimal dendroclimatic knowledge of SESE, although thin rings corresponded to extreme low precipitation years at sensitive locations [13]. For the Prairie Creek chronology, ring width correlated positively with July temperature and precipitation [19]. These findings underscore the dendroclimatic potential of SESE, which has yet to be assessed across its geographic distribution. Isotopic analyses of ∼50 years of carbon and oxygen in tree-ring cellulose revealed correlations with maximum summer temperature, hinting that isotope-based climate reconstructions may be possible in SESE [20]. Any long-term reconstructions, however, will depend upon properly crossdated tree-ring series.

Our goals in this study were to develop tree-ring chronologies for SESE and SEGI throughout their geographic distribution in California and to assess their synchrony and climatic sensitivities. While positions higher on trunks of co-dominant trees may have more interpretable annual rings, access to such locations is limited by both the large stature and protected status of remaining old-growth forests. Use of downed trees is complicated by the unknown final year of growth to anchor crossdating. We employed rope-based tree climbing to overcome these difficulties in 14 forest reserves. Within-tree replication at multiple heights along the trunk facilitated crossdating and provided a basis for comparison among locations and across species. Here we focus on inter-annual variation in radial growth to assess climatic sensitivities. Specifically, we 1) describe crossdating success with an emphasis on SESE, 2) investigate site-to-site synchrony of the tree-ring signals within and between SESE and SEGI and with other tree species, and 3) quantify correlations between ring width and climate variation, including temperature, precipitation, PDSI (Palmer Drought Severity Index), and summer cloudiness during recent time series >100 years.

## Methods

### Study Area

**Figure 2:**
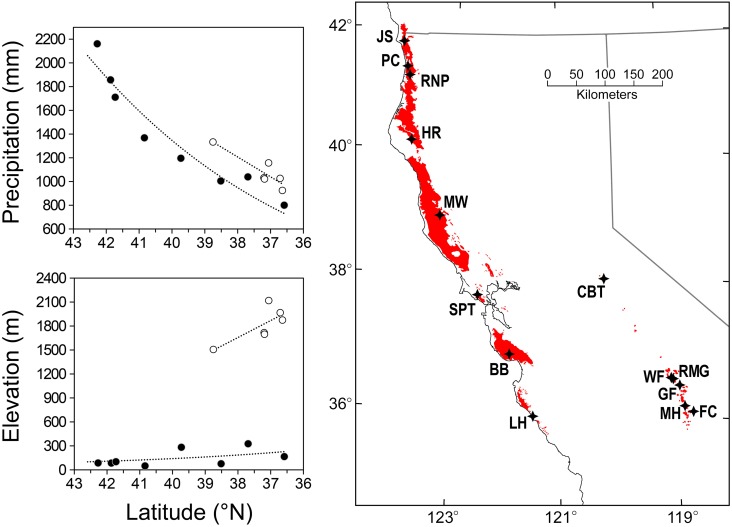
Average annual precipitation, elevation, and geographic distribution of 14 locations for two redwood species. Filled circles are *Sequoia sempervirens* locations and open circles are *Sequoiadendron giganteum* locations. Precipitation values for each location are 114-year averages (1895–2008) at 800-m resolution using PRISM data [36]. Red-shaded areas indicate native ranges of *Sequoia sempervirens* (left) and *Sequoiadendron giganteum* (right), and stars denote sampling locations.

Both redwood species grow within restricted ranges predominantly in California (Figure 2). SESE occurs along a narrow belt from its northern extent in far southwest Oregon to its southern extent along the Big Sur coast. A Mediterranean climate of wet winters and dry summers typifies this region (Figure S1) with coastal clouds contributing to SESE’s annual hydrologic input via fog drip [21] and foliar absorption [22]. Precipitation generally increases with elevation and latitude (Figure 2). Northern rainforests support the largest, oldest, and most structurally complex trees, while drier southern forests are more prone to tree-killing fires leading to a younger age distribution [23]. SEGI occurs in a narrow belt of naturally distinct groves along the western slope of the Sierra Nevada generally between 1,525 and 2,290 m with access to ample soil moisture and most groves located in the southern third of its range [24], [25]. Similar to SESE, the climate is Mediterranean but with a greater temperature range and most precipitation falling as snow between December and March [26]. Study locations spanned the native distributions of both species from north to south in high-productivity, old-growth forests exhibiting minimum human disturbance, except the partially logged Whitaker Forest (Table 1). Eight SESE sites spanned 670 km and six SEGI sites spanned 279 km (Figure 2).

**Table 1:** Characteristics and tree-ring sampling intensity for two species at 14 locations.

Species	Location	Latitude (°N)	Longitude (°W)	No. trees	No. series	No. rings
SESE	JS, Jedediah Smith Redwoods State Park	41.8	124.1	8	87	34,373
SESE	PC, Prairie Creek Redwoods State Park	41.4	124.0	9	106	37,943
SESE	RNP, Redwood National Park	41.2	124.0	14	143	71,119
SESE	HR, Humboldt Redwoods State Park	40.3	123.9	22	298	49,947
SESE	MW, Montgomery Woods State Natural Reserve	39.2	123.4	5	60	20,025
SESE	SPT, Samuel P. Taylor State Park	38.0	122.7	5	49	10,736
SESE	BB, Big Basin Redwoods State Park	37.2	122.2	5	55	13,128
SESE	LH, Landels-Hill Big Creek Reserve	36.1	121.6	8	66	13,259
SEGI	CBT, Calaveras Big Trees State Park	38.2	120.2	5	63	27,249
SEGI	WF, Whitaker Forest	36.7	118.9	19	266	57,390
SEGI	RMG, Redwood Mountain, Kings Canyon National Park	36.7	118.9	4	43	28,466
SEGI	GF, Giant Forest, Sequoia National Park	36.6	118.7	6	78	51,393
SEGI	MH, Mountain Home State Demonstration Forest	36.2	118.7	5	58	36,011
SEGI	FC, Freeman Creek, Sequoia National Monument	36.1	118.5	5	94	32,673

### Ethics Statement

Research was conducted in Jedediah Smith Redwoods State Park, Prairie Creek Redwoods State Park, Redwood National Park, Humboldt Redwoods State Park, Montgomery Woods State Natural Reserve, Samuel P. Taylor State Park, Big Basin Redwoods State Park, Landels-Hill Big Creek Reserve, Calaveras Big Trees State Park, Whitaker Forest, Kings Canyon National Park, Sequoia National Park, Mountain Home State Demonstration Forest, and Sequoia National Monument (Table 1). We obtained necessary permits from California Department of Parks and Recreation, Redwood National Park, Sequoia and Kings Canyon National Parks, and Sequoia National Monument.

### Tree Selection and Core Sampling

A total of 76 SESE and 44 SEGI trees (Table 1) were selected for sampling. The majority of study trees occurred within 16 permanent plots located in undisturbed forests containing some of the largest and most structurally complex trees at each location. In each plot, the tallest tree plus shorter and suppressed trees were climbed and measured. Additional trees from nearby forests were included so that the complete dataset contained the full range of tree sizes, crown structures, and canopy positions occurring in old-growth forests of both species, excluding trees unsafe to climb. Tree heights varied from 18.1 to 115.7 m for SESE and 26.1 to 96.3 m for SEGI. Minimum age calculations ranged from 110 to 2,510 years for SESE and 40 to 3,240 years for SEGI (Sillett et al., unpublished data). We also sampled two recently fallen trees to increase sample size at the southernmost SESE location (LH).

We climbed trees with low impact rope techniques (i.e., no spikes) and used 24″ and 32″ increment borers to collect 1–7 cores around trunk circumference at regular height intervals (about every 10 m). The number of cores collected and positions sampled varied depending on trunk size and evidence of past injuries or anomalous growth. Some cores reached beyond pith to include growth from the opposite side equating to two series (i.e., full or partial radii) per core. The lowest samples were collected above basal buttressing, typically no lower than 5 m above ground level, to avoid irregular growth and creating lower trunk wounds that weep persistently in these species (Sillett, personal observation). All cores were collected between 2005 and 2012.

### Crossdating Techniques

Cores were glued along shallow grooves in wooden mounts and polished with a sandpaper progression from 220 to 1,500 grit. We inspected each core under a stereo microscope and further polished sections with tight or wedged rings, which often revealed micro-rings along sides of SESE cores. We scanned all cores at ≥1,200 dpi and measured growth rings to the nearest 0.001 mm using WinDendro [27]. Microscope work was the primary source for deciphering growth rings, and WinDendro was used as the measuring tool.

Cores were crossdated by visually identifying marker years and specific ring-width patterns. Narrow rings provided the primary visual markers; however, large growth rings and rings with thick latewood were also used, especially in SESE. Marker years and patterns were recorded using the list method of crossdating [28]. We used COFECHA as a quality control program to confirm crossdating via correlations and to generate skeleton plots [29]. Crossdating occurred independently of previously published chronologies or marker years and was first accomplished at the tree-level and then confirmed at individual locations.

**Figure 3:**
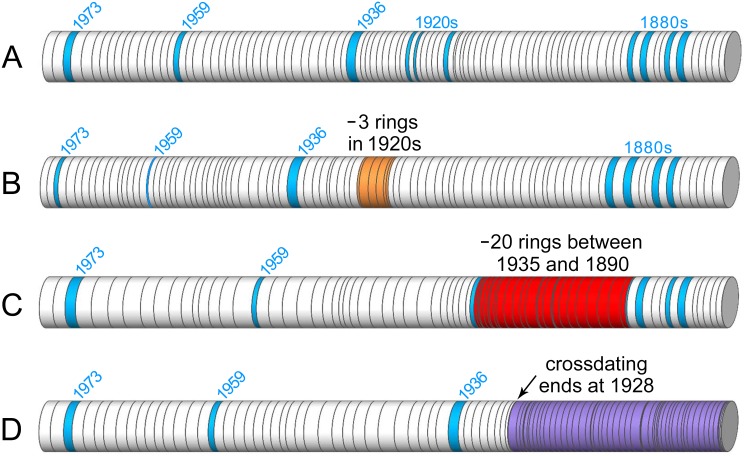
Classification system for crossdating confidence based on annual resolution. Marker years are denoted in blue. Examples are drawn from *Sequoia sempervirens* ring-width data. (A) *High crossdating confidence* or continuous annual resolution with no or few missing rings. (B) *Moderate crossdating confidence* (orange). Although missing rings are placed in their most likely location, alternative positions are possible (e.g., 3 missing rings between 1918–1926). Also assigned to sections of miniscule growth (e.g., missing rings among micro-rings). (C) *Bounded with no annual resolution* (red). Total number of missing rings in a section deduced from surrounding crossdating, but there is no indication of their most likely placement, often due to many missing rings (e.g., 20 missing rings between 1890–1935). (D) *Crossdating cessation* (purple). No annual resolution for a section where crossdating terminated and interior rings are neither resolved nor bound by known markers (e.g., crossdating progressed towards pith and ceased at 1928, leaving an undated section of core with a minimum ring count).

Given SESE’s tendency for radial growth irregularities, care was taken in determining final dates. Each tree’s chronology was initially built upon the best series (e.g., series with distinct ring boundaries, strong marker years, and limited sections of extremely narrow or wedged rings). Strong correlations in COFECHA (*r*≥0.32, *P*<0.01) verified inclusion of these series in a preliminary chronology. Missing rings were located based on visual crossdating with other cores from the same tree, morphological characteristics of adjacent rings (e.g., wedging, narrow rings, or damaged cells), and confirmation in COFECHA. Series with large sections of difficult rings were floated as unknowns against the master chronology in COFECHA to identify possible dates for earlier years to examine visually. To manage complex series, we classified rings into four categories based on degree of annual resolution achieved: 1) *high crossdating confidence*, 2) *moderate crossdating confidence*, 3) *bounded with no annual resolution*, and 4) *crossdating cessation* (Figure 3).

### Crossdating Statistics

Patterns of crossdating success (*high* plus *moderate confidence*) and missing rings were assessed for each species at the tree level. Differences among species were confirmed using Levene’s test for homogeneity of variance and ANOVA with robust Tukey contrasts [30]. The same method was applied using suppression status and spiral compression wood as categorical variables to help explain some of the variation in crossdating success within SESE trees. Suppressed trees were those overtopped by neighbors or heavily shaded on their southern flank. Spiral compression wood was assigned to trees if the pattern was exhibited by at least one core.

Core-level data were used to compare within-tree crossdating variability for each species and to assess the degree of difficulty in crossdating basal versus non-basal cores in SESE. We calculated the standard deviation among cores for each tree and used ANOVA with robust Tukey contrasts to test for significant differences. To test the assumption that basal cores were harder to crossdate than other cores in SESE, a paired Wilcoxon signed-rank test was applied to the mean proportion of crossdated rings of the lowest position versus all higher positions on each tree. All crossdating analyses were performed in R [31].

### Creation and Synchrony of Chronologies

We created a standardized chronology at each site to present a template for crossdating and investigation of inter-annual climate sensitivity. First, we created tree-level chronologies, including all series from a given trunk >50 years with *high crossdating confidence*. We used ARSTAN [32] to standardize ring widths around a dimensionless index of 1.0 and applied a 32-year cubic smoothing spline to remove the age-related geometric growth trend and other low-frequency variation. This approach optimized expression of high-frequency variation most useful for crossdating and suitable for inter-annual climate analysis [33]. Tree-level chronologies (standard version) were then combined into site-level chronologies with no further detrending. Standard versions of site chronologies were used for crossdating and identification of marker years, whereas residual versions, which removed first-order autocorrelation and stabilized variance [34], were used for analyses of synchrony and climate sensitivity.

Synchrony of chronologies was assessed by comparing marker years among locations and by correlation analysis. Marker years were categorized using the 10 smallest standardized ring widths per century, and years with three or fewer locations in common were removed to identify the strongest regional markers. We used correlation analysis to compare the common variance among residual chronologies over the well-replicated time period 1750–2008. To evaluate how correlations between these chronologies changed with distance, we computed Euclidean distance between each pair of locations using UTM coordinates and elevations.

### Regional Assessment

We used correlation analysis to compare inter-annual variability of SESE and SEGI radial growth with that of other tree species. Species chosen on the basis of proximity to study locations, known climate sensitivities, and chronology length included *Pseudotsuga menziesii* (PSME), *Chamaecyparis lawsoniana* (CHLA), *Juniperus occidentalis* (JUOC), *Pinus ponderosa* (PIPO), *Quercus douglasii* (QUDG), and *Pinus jeffreyi* (PIJE). Tree-ring data were accessed via the International Tree Ring Data Bank [35] except the Oregon PSME chronologies (Bryan Black, unpublished data). Raw ring-width series were subjected to the same steps of chronology creation described in the previous section. SESE and SEGI chronologies were collapsed into regional chronologies represented by northern SESE sites (JS, PC, RNP, HR), the southernmost SESE site (LH), and all SEGI sites. The maximum common period of 1760–1980 was used in these analyses.

### Climate and Radial Growth

Monthly maximum temperature, minimum temperature, and precipitation data were obtained for each location using the PRISM (Parameter-elevations Regressions on Independent Slopes Model) climate mapping system with 800 m resolution [36]. PRISM uses point measurements, a digital elevation model, knowledge of geospatial climatology, and considers both topography and proximity to the Pacific Ocean [37], [38]. Monthly precipitation as snow at 4 km resolution was acquired for each SEGI location from Climate Western North America (ClimateWNA), which relies on PRISM data for a baseline [39].

Palmer Drought Severity Index (PDSI), which incorporates the effects of temperature, precipitation, and evapotranspiration [40], provided a proxy for soil moisture availability. PDSI utilizes a water balance model where positive values reflect wet conditions and negative values reflect dry conditions. Monthly location-specific PDSI as well as California and regional summer (average June–September) PDSI data were used to assess growth sensitivities. Monthly PDSI data at 4 km resolution were obtained from Western Regional Climate Center’s WestWide Drought Tracker, which uses both PRISM and North American Land Data Assimilation System Phase 2 data [41]. State and regional PDSI data were obtained from the National Climatic Data Center, using regions CA01 (North Coast California) for all SESE north of the San Francisco Bay, CA04 (Central Coast California) for all SESE south of San Francisco Bay, and CA05 (Sierra Nevada) for all SEGI.

We used reconstructed northern California 1901–2008 airport fog (i.e., cloud base ≤400 m elevation) derived from Arcata and Monterrey airport data (1951–2008) and the inland-coast difference in maximum temperature [42]. A 32-year spline was applied to detrend these data and emphasize inter-annual variation. Average June–September airport fog was used to express summer cloudiness in SESE sites, all of which occurred below 400 m elevation (Figure 2).

Relationships between monthly climate and residual chronologies were examined with combined bootstrapped Pearson’s correlation and response function analyses (RFA) for the period 1895–2008 using the bootRES package in R [43]. RFA removed multicollinearity between months before assessing dendroclimatic relationships [43]. Significant relationships revealed by both Pearson’s correlations and RFA were emphasized. To account for potential effects of the previous growing season on current radial growth, our analysis window included the beginning of previous through end of current growing season (March–October for SESE; May–October for SEGI). We also analyzed correlations between residual chronologies and both summer cloudiness (1901–2008) and summer PDSI (1895–2008).

## Results

### Crossdating

We analyzed 76 trees, 864 series, and 250,530 growth rings at eight locations spanning the years 328–2012 for SESE and 44 trees, 602 series, and 233,182 growth rings at six locations spanning the years 474–2012 for SEGI (Table 1). All rings were classified by annual resolution: 1) *high crossdating confidence*, 2) *moderate crossdating confidence*, 3) *bounded with no annual resolution*, and 4) *crossdating cessation* (Figure 3). We crossdated 82.6% of SESE rings (76.4% *high confidence* and 6.2% *moderate confidence*), compared to 99.4% of SEGI rings (98.9% *high confidence* and 0.5% *moderate confidence*). Missing rings represented 4.4% of SESE rings, which was a minimum estimate; the *crossdating cessation* category (7.9% of rings) was not included in the calculation, and this category had higher proportions of tight and likely missing rings. By comparison, SEGI was missing only 0.2% of rings and had only 0.3% of rings in the *crossdating cessation* category. Classification of SESE rings in the *bounded without annual resolution* category (9.4%) often occurred in series with many missing rings. Tight rings were more common in SESE with 8.2% of widths <0.25 mm and 2.4% <0.10 mm, compared to 6.3% and 0.7% for SEGI respectively.

Tree-level proportions of crossdated and missing rings differed between species. The proportion of crossdated rings was 15% lower in SESE than SEGI (*P*<0.001). The range of tree-level crossdating success was also considerably larger in SESE, 3.7–100% (mean = 84.9), compared to 89.7–100% (mean = 99.5) in SEGI. While many trees of both species had all rings annually resolved, 71% of SESE trees compared to 20% of SEGI trees had less than perfect crossdating. Within SESE, both suppressed trees and those exhibiting spiral compression wood had a lower percentage of crossdated rings (*P* = 0.030 and *P* = 0.007, respectively). Suppressed trees averaged 66.6% crossdated rings compared to 87.9% in unsuppressed trees, and trees with spiral compression wood averaged 60.3% crossdated rings compared to 88.1% in trees without this trait. The tree-level proportion of missing rings was higher and varied more in SESE than SEGI (*P*<0.001). For SESE and SEGI respectively, the mean proportion of missing rings per tree was 3.1% versus 0.1% and ranged from 0–12.5% versus 0–0.9%.

Within-tree variation in crossdating differed between species. The standard deviation of percent crossdated rings among cores from the same tree was greater (*P*<0.001) in SESE (range 0–48.3, mean 14.3) than SEGI (range 0–13.4, mean 1.0). Higher variation in SESE was attributable, in part, to poorer crossdating of lowest trunk cores, which crossdated an average of 10.6% less often than cores higher up trunks (*P*<0.001).

### Chronology Characteristics and Synchrony

**Figure 4:**
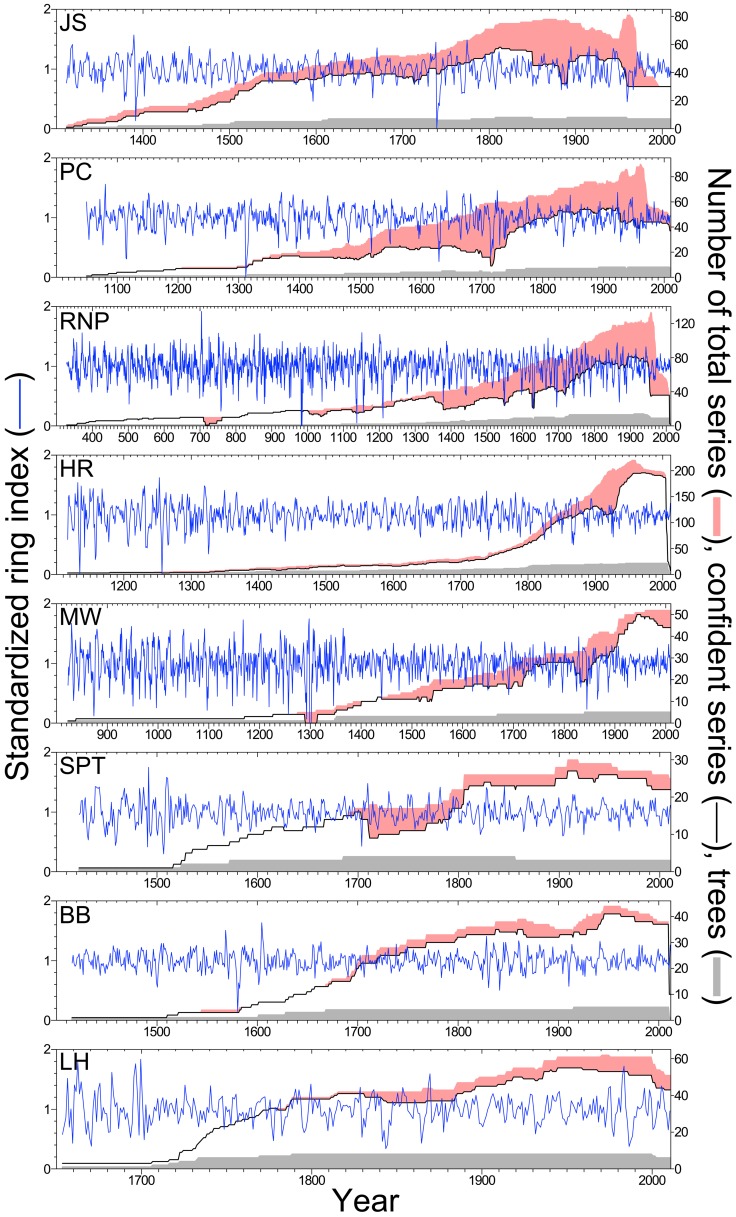
Standardized tree-ring chronologies and sample depths for eight *Sequoia sempervirens* locations. Blue lines indicate ring-width indices for each location, using series >50 years in length with *high crossdating confidence* and detrended with a 32-year cubic smoothing spline. Pink shading denotes difference between total number of series sampled and number of series with *high crossdating confidence*. Tree sample sizes indicated in gray.

**Figure 5:**
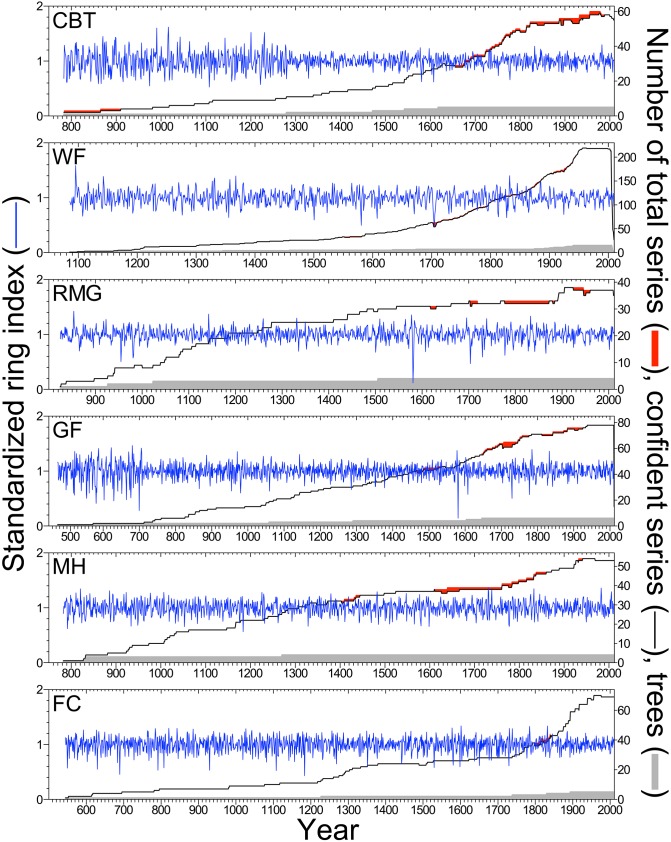
Standardized tree-ring chronologies and sample depths for six *Sequoiadendron giganteum* locations. Blue lines indicate ring-width indices for each location, using series >50 years in length with *high crossdating confidence* and detrended with a 32-year cubic smoothing spline. Red shading denotes difference between total number of series sampled and number of series with *high crossdating confidence*. Tree sample sizes indicated in gray.

We developed crossdated tree-ring chronologies for all 14 locations using series with *high crossdating confidence* detrended with a 32-year spline (Table S1). SESE chronologies spanned 1,685 years (328–2012) and were longer for middle and northern latitude locations where older trees persist **(** Figure 4). SEGI chronologies spanned 1,538 years (474–2011) with all chronologies reaching to at least 1085 (Figure 5). Several statistics described crossdating strength and chronology quality, highlighting the need for more tree replication in SESE locations (Figure 4, Table 2). Years with a greater difference between possible and actual series indicated less successful crossdating, often associated with missing or extremely narrow rings. Crossdating success among series was uniformly high for all SEGI chronologies (Figure 5). The dip in replication for the most recent years was caused by selecting only *highly confident* series >50 years long, and high ring-width variation apparent early in the chronologies was attributable to low sample size [44]. Chronologies at each location were established independently except for the years 1289–1313 at MW where placement of six missing rings was based on the HR chronology. Chronologies exhibited reasonable quality and signal strength given the low tree replication (Table 2).

**Table 2:** Statistical characteristics of standard tree-ring chronologies for two species at 14 locations.

Species	Location	Years	1 tree cutoff	Mean 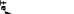	Interseries 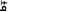	Unfiltered auto- 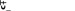	Between trees rbar^d^ *	EPS^e^ *	No. trees common period*
SESE	JS	1311–2009	1370	0.288	0.554	0.773	0.530	0.887	7
SESE	PC	1049–2010	1313	0.289	0.489	0.809	0.403	0.844	8
SESE	RNP	328–2012	1093	0.277	0.529	0.797	0.370	0.804	7
SESE	HR	1071–2011	1326	0.247	0.601	0.762	0.454	0.924	16
SESE	MW	822–2009	1347	0.277	0.634	0.804	0.448	0.803	5
SESE	SPT	1422–2010	1523	0.286	0.580	0.805	0.627	0.834	3
SESE	BB	1415–2011	1600	0.220	0.585	0.737	0.472	0.781	4
SESE	LH	1653–2010	1705	0.323	0.725	0.695	0.472	0.843	6
SEGI	CBT	783–2009	1278	0.182	0.564	0.765	0.459	0.809	5
SEGI	WF	1085–2009	1098	0.182	0.610	0.767	0.455	0.893	10
SEGI	RMG	824–2011	925	0.176	0.566	0.822	0.549	0.829	4
SEGI	GF	474–2011	712	0.187	0.629	0.797	0.509	0.862	6
SEGI	MH	782–2011	830	0.169	0.676	0.774	0.643	0.900	5
SEGI	FC	544–2010	1226	0.159	0.640	0.729	0.453	0.805	5

a Measure of year-to-year variability of annual rings [46].

b Average correlation of every series to the master (excluding series in question) [46].

c Reflects prior year’s influence on current year’s growth [46].

d Correlation between tree-ring chronologies, representing strength of common variability [85].

e Degree to which the chronology represents a hypothetical perfect chronology. Function of rbar and sample size, with 0.85 as a recommended cutoff for confidence [44], [85].

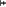
Average tree-level statistics, using series >50 years with high and moderate crossdating confidence. Calculated using COFECHA.

*Common period 1901–2000.

Marker years in both species aided crossdating but in a different manner. For SESE, crossdating was complex with frequent missing, tight, wedged, and abnormal rings (mean tree-level sensitivity = 0.273), which had to be confirmed nearly every decade by marker years. Accordingly, crossdating SESE involved recognizing not only low-growth marker years (e.g., 1824, 1924), but also years with consistently large ring widths (e.g., 1936, 1983), signature ring-width patterns (e.g., small 1883, 1887, and 1889), rings with distinctively thick latewood (e.g., 1923), and growth reductions (i.e., several years of sustained low-growth rings) (e.g., 1865–1869). Of the eight low-growth SESE years listed by Schulman [13] for the time period 1824–1924, five were in the lowest decile of ring widths at MW, about 20 km away. For SEGI, intermittent low-growth marker years approximately every few decades guided crossdating among rings with similar widths (mean tree-level sensitivity = 0.178) and infrequent missing rings. Seventy percent of marker years listed by Hughes and Brown [6] common to the lowest decile of their three SEGI sites (GF, MH, and Camp Six) were among the lowest decile of ring widths for our unified SEGI chronology (sample period of 782–1988 with three-site replication).

**Figure 6:**
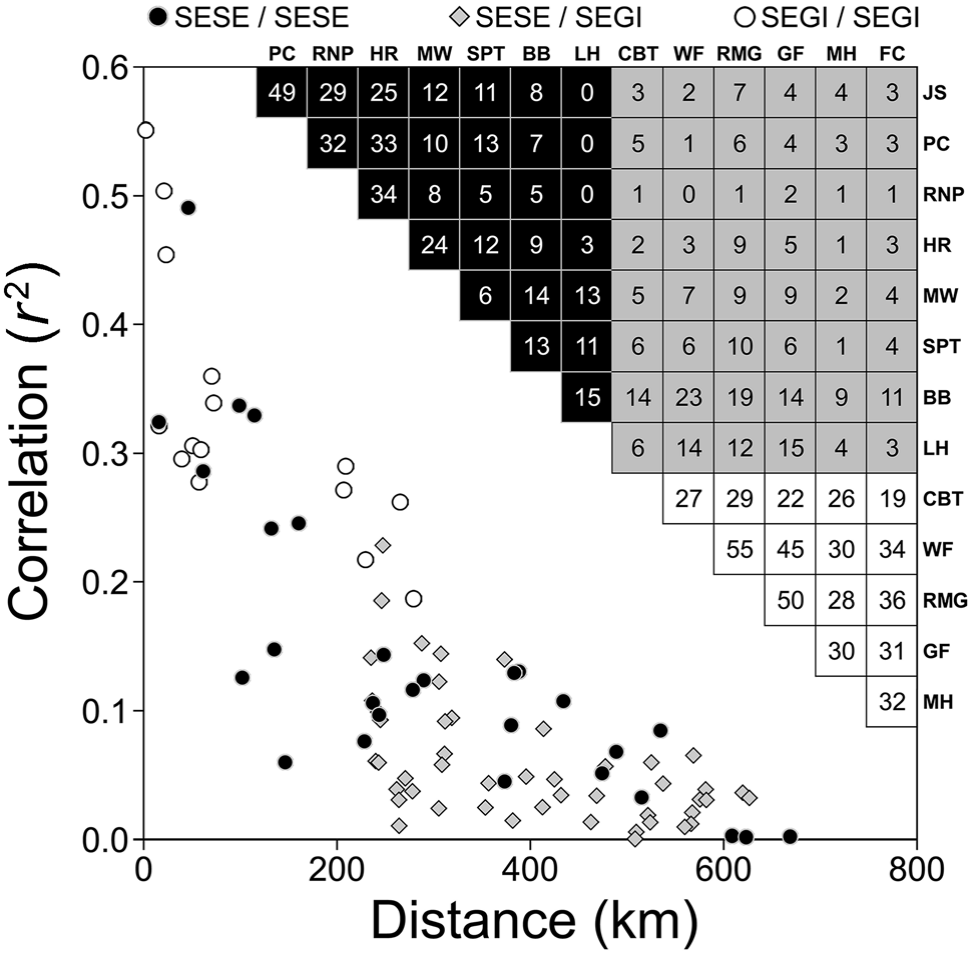
Correlations among redwood tree-ring chronologies and relationships with Euclidean distances between locations. Values in cells are % variance explained (100×*r* ^2^) for pairwise correlations over a 259-year common period (1750–2008). Locations arranged by latitude from north (left and top) to south (right and bottom) within species.

Common marker years and patterns of inter-annual variation emerged at the regional scale (Figure S2). Several marker years were common to both species (e.g., 1500, 1598, 1729, 1865, 1924, 1959), while others clustered by species (e.g., 1739, 1742, 1850 for the northern SESE and 1580, 1637, 1733, 1827, 1829, 1841 for SEGI). Southern SESE locations often shared strong marker years more frequently with SEGI than northern SESE locations (e.g., 1580, 1733). The common climate response indicated by shared marker years was reflected in the synchrony of residual chronologies, which was clearly stratified by distance (Figure 6). Northern locations (JS, PC, RNP, HR) correlated strongly with each other (*P*<0.0001), while southern SESE locations (BB, LH) correlated more strongly with SEGI than with northern SESE locations. Indeed, the southernmost SESE chronology (LH) was virtually independent of northern SESE chronologies. All SEGI chronologies correlated significantly (*P*<0.0001) with each other, but the most northern and isolated location (CBT) had the weakest correlations.

### Regional Assessment

Common ring-width variation between redwoods and other species reflected spatial synchrony of climate forcing (Table 3). The northern SESE chronology correlated most strongly with the CHLA chronology from the nearby Klamath-Siskiyou Mountains of southwestern Oregon. Of the PSME chronologies, northern SESE co-varied most with the two coastal Oregon chronologies and correlated more with PSME from the Olympic Peninsula of Washington than with PSME from Point Reyes, California. Moreover, the northern SESE chronology correlated more consistently with PIPO, JUOC, PIJE, and QUDC chronologies from northern California than with those from the Sierra Nevada and central California. The southernmost SESE chronology (LH) showed consistently strong correlations with QUDG chronologies throughout California, especially those from the central California Coast Range. The only PSME chronology strongly correlated with the LH chronology was from Point Reyes. The LH chronology also correlated strongly with PIJE chronologies of the southern Sierra Nevada and exhibited weaker correlations with PIPO, JUOC, and PIJE chronologies of northern California. The unified SEGI chronology showed consistent, positive correlations with tree-ring chronologies throughout California as well as the CHLA chronology from southwestern Oregon.

**Table 3:** Correlations between tree-ring indices of SESE and SEGI and six other western North American tree species.

Species	Location	Latitude (°N)	Longitude (°W)	Elevation (m)	State	Northern SESE (*r*)	Southernmost SESE (*r*)	Unified SEGI (*r*)	Contributor
PSME	Olympic Road	48.00	−124.00	267	WA	**0.22**	−0.03	0.06	Earle et al.
PSME	Newport	44.40	−124.03	20	OR	**0.38**	0.09	**0.19**	Black
PSME	Cape Perpetua	44.30	−124.07	244	OR	**0.29**	−0.01	0.12	Black
PSME	Fryday Ridge	40.75	−123.67	1560	CA	**0.19**	−0.10	**0.27**	Briffa & Schweingruber
PSME	Pt. Reyes	38.02	−122.80	120	CA	0.12	**0.28**	**0.22**	Brown
CHLA	Page Mountain	42.00	−123.57	1070	OR	**0.40**	−0.06	**0.42**	Carroll & Jules
JUOC	Sharp Mountain	41.72	−121.80	1417	CA	**0.29**	**0.38**	**0.40**	Holmes et al.
JUOC	Carson Pass East	38.70	−120.00	2591	CA	0.12	0.06	**0.39**	Meko et al.
JUOC	Kaiser Pass	37.28	−119.08	2731	CA	**0.20**	**0.24**	**0.42**	Holmes et al.
PIPO	Damon’s Butte	41.50	−121.17	1448	CA	**0.19**	**0.33**	**0.48**	Graumlich
PIPO	Grizzly Peak	41.17	−122.03	1463	CA	**0.19**	−0.01	**0.30**	Graumlich
PIPO	St. Johns Mountain	39.43	−122.68	1555	CA	0.15	0.05	**0.34**	Holmes
QUDG	Dibble Creek	40.42	−122.63	218	CA	0.12	**0.47**	**0.30**	Stahle et al.
QUDG	Eel River	39.82	−123.07	610	CA	**0.17**	**0.39**	**0.44**	Stahle et al.
QUDG	Putah Creek	38.67	−122.45	180	CA	0.01	**0.60**	**0.33**	Stahle et al.
QUDG	Mt. Diablo	37.87	−121.95	245	CA	0.00	**0.66**	**0.33**	Stahle et al.
QUDG	North Fork Kaweah River	36.92	−118.90	701	CA	**0.16**	**0.56**	**0.37**	Stahle et al.
QUDG	Pinnacles National Monument	36.47	−121.18	350	CA	−0.05	**0.67**	**0.23**	Stahle et al.
PIJE	Blue Banks	39.67	−122.97	1598	CA	**0.20**	−0.08	**0.31**	Holmes et al.
PIJE	Buena Vista	36.72	−118.88	2280	CA	0.15	−0.02	**0.39**	Holmes et al.
PIJE	Kennedy Meadows	36.03	−118.18	2024	CA	0.01	**0.48**	**0.34**	Holmes et al.
PIJE	Piute Mountain	35.53	−118.43	1975	CA	0.15	**0.47**	**0.47**	Holmes et al.

Product-moment correlations (r) for the common period 1760 to 1980, with statistically significant correlations (r≥0.16, P<0.01) highlighted in bold.

Species: PSME, Pseudotsuga menziesii (Douglas-fir); CHLA, Chamaecyparis lawsoniana (Port Orford cedar); JUOC, Juniperus occidentalis (Western juniper); PIPO, Pinus ponderosa (ponderosa pine); QUDG, Quercus douglasii (blue oak); PIJE, Pinus jeffreyi (Jeffrey pine).

Locations listed in descending order of latitude by species. States: WA, Washington; OR, Oregon; CA, California.

Northern SESE = JS, PC, RNP, HR; Southernmost SESE = LH; Unified SEGI = all SEGI locations.

All chronologies accessed via International Tree Ring Data Bank, except Black’s two unpublished PSME chronologies.

### Dendroclimatic Relationships

**Figure 7:**
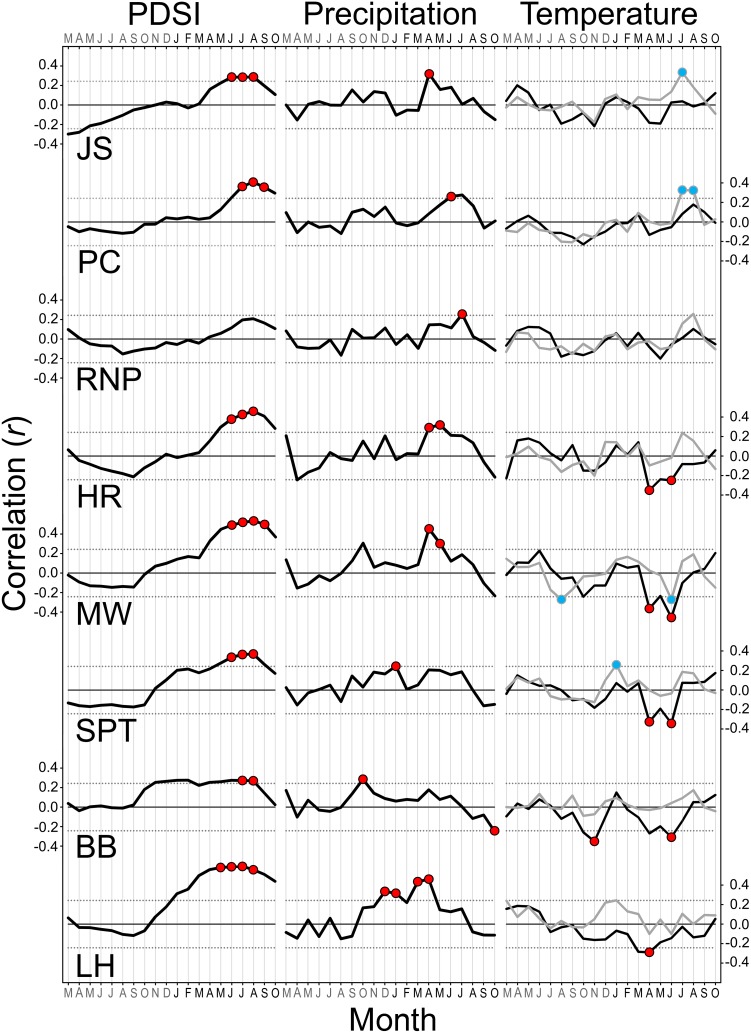
Summary of climate sensitivities for eight *Sequoia sempervirens* locations. Bootstrapped correlations and response functions of residual tree-ring chronologies against monthly Palmer Drought Severity Index (PDSI), precipitation, and maximum and minimum temperature conducted over a 114-year common period (1895–2008). Thicker black lines indicate correlation values. Thin dotted lines represent cutoff for statistical significance (*P*<0.01). Colored circles show months with significant correlations and response functions. Grey lines and blue circles represent minimum temperature. Letters indicate 20 months from March of previous year to October of current year. Locations arranged by latitude from north (top) to south (bottom).

**Figure 8:**
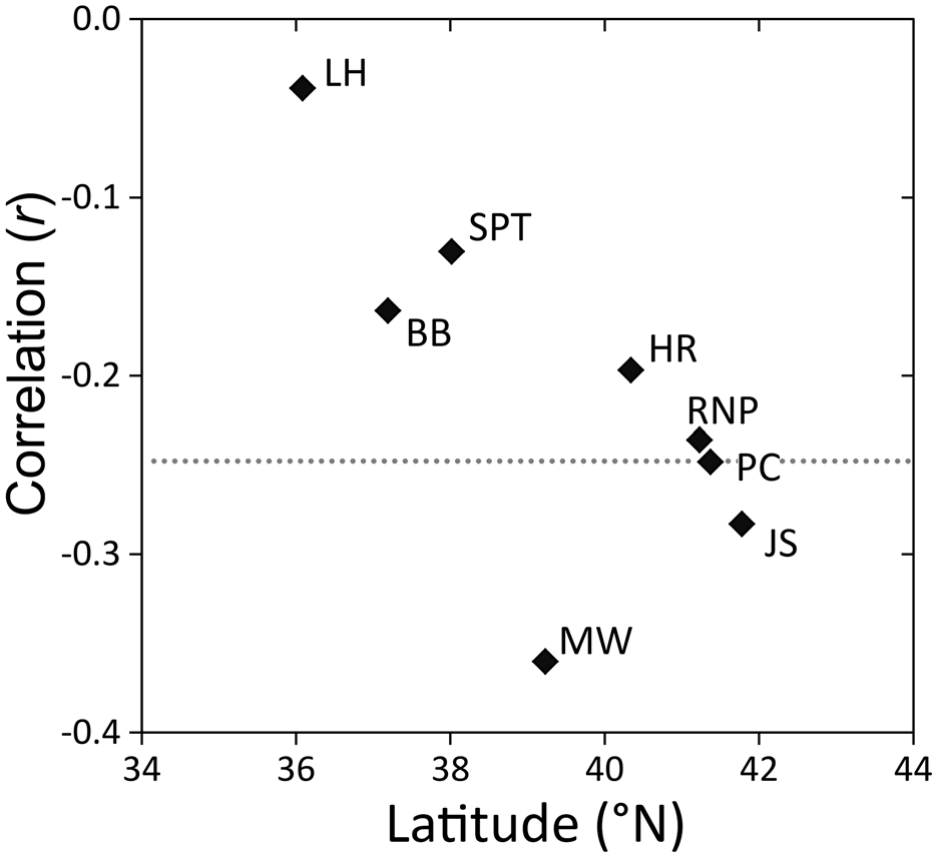
Correlations between residual tree-ring chronologies and reconstructed summer cloudiness for eight *Sequoia sempervirens* locations. Summer cloudiness (i.e., airport fog with cloud base ≤400 m elevation) reconstructed by Johnstone and Dawson [42] for June–September over a 108-year common period (1901–2008). Dotted line represents cutoff for statistical significance (*P*<0.01).

Consistent relationships between monthly climate and residual tree-ring chronologies were evident in SESE (Figure 7). PDSI during the growing season correlated positively with radial growth at all locations with only one location (RNP) lacking any significant response functions. The positive relationship between PDSI and radial growth extended earlier in the growing season at southern locations. Precipitation correlated positively with radial growth at all locations, and response functions were significant for April at JS, June at PC, July at RNP, April and May at HR and MW, January at SPT, prior October at BB, and four months during winter and spring at LH. Temperatures correlated with radial growth at all locations with only one location (RNP) lacking any significant response functions. Radial growth in the two northernmost locations (JS, PC) increased significantly with minimum July temperature (and August at PC) as did radial growth with minimum January temperature at SPT. The only significant negative responses to minimum temperature occurred at MW (July and prior August). Radial growth decreased significantly with maximum temperature in April and/or June at all locations from HR south to LH, as did radial growth with maximum prior November temperature at BB. SESE radial growth increased with decreasing summer cloudiness (i.e., airport fog), and correlations were significant (*P*<0.01) at three locations (JC, PC, MW) (Figure 8). The relationship between radial growth and summer cloudiness generally weakened towards the south, but inland and centrally located MW exhibited the strongest correlation (*P* = 0.0001).

**Figure 9:**
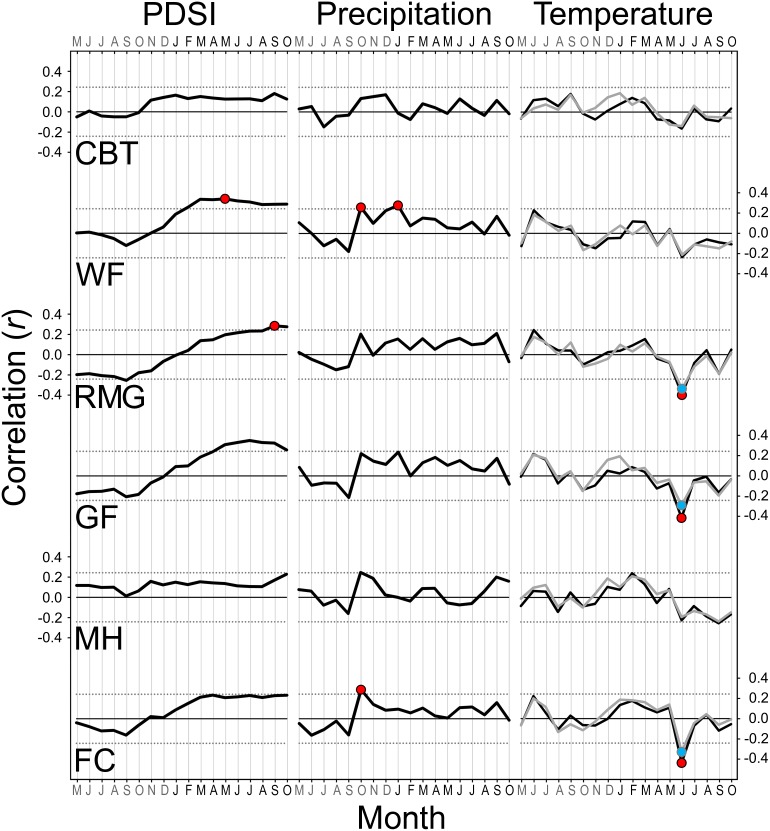
Summary of climate sensitivities for six *Sequoiadendron giganteum* locations. Bootstrapped correlations and response functions of residual tree-ring chronologies against monthly Palmer Drought Severity Index (PDSI), precipitation, and maximum and minimum temperature conducted over a 114-year common period (1895–2008). Thicker black lines indicate correlation values. Thin dotted lines represent cutoff for statistical significance (*P*<0.01). Colored circles show months with significant correlations and response functions. Grey lines and blue circles represent minimum temperature. Letters indicate 18 months from May of previous year to October of current year. Locations arranged by latitude from north (top) to south (bottom).

Significant dendroclimatic patterns were relatively few in SEGI (Figure 9). Growing season PDSI correlated positively with radial growth at all locations, but only two response functions were significant (May PDSI at WF, September PDSI at RMG). Precipitation also correlated positively with radial growth at all locations, but only three response functions were significant (prior October precipitation at WF and FC, January precipitation at WF). There were no significant correlations between radial growth and annual precipitation as snow, except for a positive correlation (*r* = 0.24) in January at WF. June maximum and minimum temperatures correlated negatively with radial growth at all locations, and these response functions were significant at RMG, GF, and FC.

**Figure 10:**
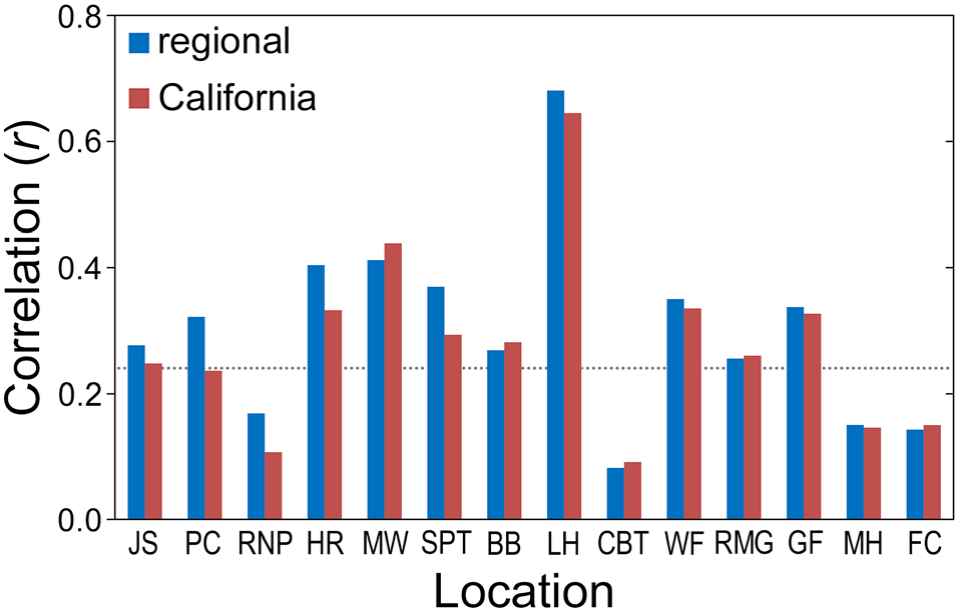
Summary of radial growth sensitivity to summer drought indices for two species at 14 locations. Correlations of residual *Sequoia sempervirens* and *Sequoiadendron giganteum* tree-ring chronologies against regional and state summer drought (average June–September PDSI) indices conducted over a 114-year common period (1895–2008). Dotted line represents cutoff for statistical significance (*P<*0.01). Locations arranged by latitude from north (left) to south (right) within species.

**Figure 11:**
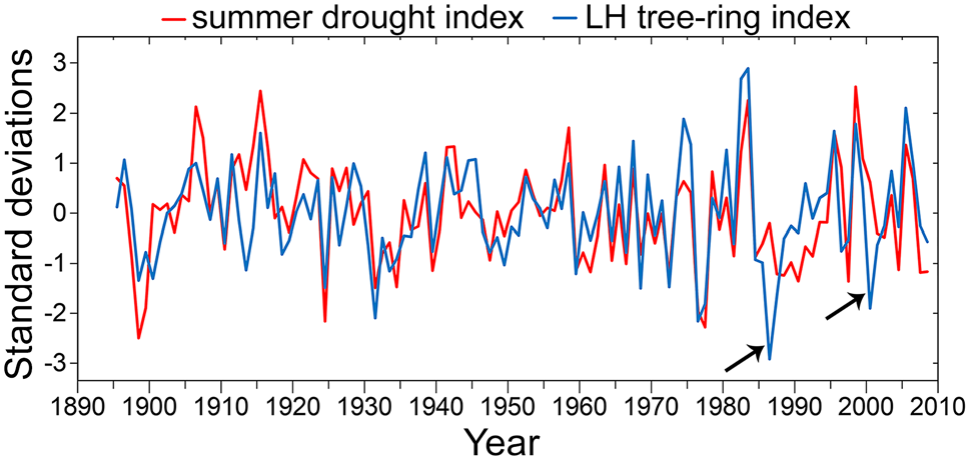
Visual comparison of southernmost *Sequoia sempervirens* (LH) tree-ring index and Central Coast California summer drought index. Summer drought is average June through September PDSI from the National Climatic Data Center for CA04 (Central Coast California). Both indices standardized by their standard deviates. Note discrepancies after known forest fires in 1985 and 1999 (arrows).

Radial growth of SESE and SEGI at all locations declined with increasing severity of summer drought, as captured by regional and state PDSI. Correlations between tree-ring chronologies and summer drought indices were statistically significant at ten locations, but by far the strongest correlation occurred at LH (Figure 10). The summer drought index for the central California coast was 46% coincident with the residual LH chronology (Figure 11).

## Discussion

### Crossdating

We successfully crossdated the majority of tree rings at all locations. Crossdating SESE was effective albeit more difficult than SEGI due to tight rings, missing rings, ring wedging, and spiral compression wood. These SESE growth anomalies were not pervasive, as some series provided continuous, visible rings spanning centuries while others had scores of missing rings (e.g., one tree at PC had 119 missing rings between 1659 and 1970). Our classification of nearly half a million rings by crossdating confidence will aid ongoing research. For example, areas that are *bounded with no annual resolution* are still useful for tree-level calculations of wood production and minimum age, while only *high crossdating confidence* will suffice for climate reconstructions and analyses of stable isotopes.

Unlike the situation in SEGI, crossdating success varied greatly among SESE trees. We confirmed previously reported difficulties crossdating suppressed SESE [13], [14] and SESE with spiral compression wood [13], [19]. Spiral compression wood is a relatively rare phenomenon [45] and may be more frequent in some portions of the SESE distribution. All nine of our study trees displaying spiral compression wood were from the three northernmost locations, though the phenomenon does occur occasionally throughout the range (Sillett, personal observation). Sampling trees of many ages facilitated millennium-scale crossdating. While younger trees often provided easy-to-decipher wider rings, older trees with tight outer rings allowed borers to sample discernible rings farther back in time. For example, a 35 m core from one SESE had 117 missing rings and *no annual resolution* from 1545–1971. This core had well-defined growth rings past this period extending to 1094 and thus provided much-needed replication for the earliest portion of the RNP chronology.

High within-tree variation in SESE crossdating success was partly attributable to more difficult lower trunk series, emphasizing the need for whole-trunk sampling. Despite avoiding abnormalities near the tree base, crossdating SESE was still more difficult in the lowest position sampled than higher on trunks, corroborating previous observations [13], [14]. Series collected from relatively low on trunks tended to have diminished crossdating success, but they sometimes had wider rings associated with buttressing, often providing access to otherwise indecipherable periods of a tree’s growth history. Growth irregularities characteristic of SESE were not limited to the lower trunk, however, and within-tree crossdating repeatedly relied on all samples collected from a given trunk. Regardless of height, certain series provided discernible rings where others did not. Sampling standing trees at multiple heights and azimuths along the trunk is therefore necessary to successfully crossdate SESE in many cases.

### Chronology Characteristics and Synchrony

We established the first range-wide network of SESE chronologies. The only previous published SESE chronology extended to 1750 [10], while our longest chronology (RNP) extended to 328 with two-tree replication to 1093. We crossdated independently, and upon review, our PC chronology correlated strongly (*r* = 0.54; *P*<0.001) with the existing chronology derived from other trees in this reserve [10]. Two-tree replication exceeded 600 years in northern and central locations (JS, PC, RNP, HR, and MW) and 300–400 years in southern locations (SPT, BB, LH) (Table 2). Given our within-tree sampling strategy, which was often necessary to attain tree-level crossdating and associated research goals, tree-level replication was generally low by dendrochronological standards [46]. We present these SESE chronologies as proof of concept for crossdating and as a fruitful foundation upon which to build. In addition to early years with low replication, years with large differences between total and confidently crossdated series warrant further sampling (e.g., 1626–1632 at RNP, Figure 4).

Our SEGI chronologies contributed to the breadth and robustness of tree-ring records for this species [4], [47], [48] and revealed commonalities with published marker years [6]. While much of the SEGI range is well-replicated, the northern extent is comparatively under-represented, and we improved it with the 1,226-year chronology at CBT. We augmented replication in the southern range with the 1,466-year chronology at FC, and we extended all SEGI chronologies well into the twenty-first century as many previous chronologies end in the early 1990s. These recent decades are important as tree-ring records provide a basis for long-term monitoring.

Range-wide chronologies permitted assessments of inter-annual climate variation across California, revealing latitudinal differentiation among SESE chronologies compared to a more unified SEGI signal. Northern SESE chronologies exhibited strong synchrony, and southern SESE chronologies often shared marker years and correlated more strongly with SEGI than northern SESE. Schulman alluded to such connections, noting that occasional small rings in SESE corresponded with SEGI marker years [49]. The distance separating distinctive northern and southern SESE tree-ring chronologies is far less than that observed in another western conifer, *Tsuga mertensiana* [50], which emphasizes the need to factor geographic location into models predicting SESE responses to climate change. While varying expressions of limiting climatic conditions were found among locations and species, SESE and SEGI chronologies often shared marker years reflecting large-scale events, such as historical (e.g., 1924) [51] and inferred (e.g., 1777, 1500) [6] droughts and El Niño years (e.g., large rings at 1982–1983) [52].

### Regional Assessment

Comparisons with other western tree species helped place our SESE and SEGI chronologies into a regional context. The southernmost SESE chronology (LH) showed synchrony with several moisture-sensitive tree species in California, particularly QUDG, which exhibits a strong cool-season precipitation signal useful for climate reconstructions [53], [54]. Indeed our LH chronology correlated slightly more than the closest QUDG chronology (Pinnacles National Monument) with annual California PDSI (*r* = 0.64 vs. *r* = 0.62) over the common period 1895–2003. Part of SESE’s value in climatic reconstructions is the potential to extend chronologies farther back in time by virtue of decay-resistant heartwood.

Northern SESE rainforests have been considered the southern extent of coniferous forests in the Pacific Northwest [55] and are ecologically similar to coastal rainforests in Washington and Oregon [23]. Indeed, the northern SESE chronology was more similar to a PSME chronology (Olympic Road) from ∼750 km away in Washington than to a PSME chronology from only ∼375 km away in California. Northern SESE locations are an important addition to the dendrochronological record, as the southern Pacific Northwest is relatively underrepresented in tree-ring archives [35], and individual locations here influence gridded tree-ring networks more than in other regions (e.g., American Southwest) [56]. Although dendroclimatic signals can be species- and site-specific, correlations between SESE and other tree-ring chronologies reflect the spatial synchrony of extreme climate years across a broad geographic area [57]–[59].

The smallest ring in the replicated northern SESE chronology occurred in 1739 (Figure 1F). In the northernmost location (JS), this year’s ring index was more than six standard deviations below the residual chronology mean for 698 years (1311–2008) (Figure 4). This ring also had the lowest index value and recorded cell damage in the CHLA chronology from southwestern Oregon [60] and was a strong marker year in PSME chronologies from coastal Oregon (Black, personal communication). Precipitation reconstruction based on six drought-sensitive conifer species in the Pacific Northwest showed 1739 as a severe, single-year drought event [57], while the more inland PDSI grid-point showed 1739 as a relatively dry, but not severe, soil moisture year [61]. Strength of the 1739 climate signal in tree-ring chronologies from northwestern California and southwestern Oregon highlights this region’s distinctive climate.

The smallest ring in the unified SEGI chronology occurred in 1580 (Figure 1G) and has a well-documented regional extent. Of 22 SEGI trees with cores deep enough to reach this year, seven yielded at least one core missing 1580. The most extreme situation occurred at the highest elevation location (GF), where this year’s ring index was more than six standard deviations below the residual chronology mean for 1538 years (474–2011) (Figure 5). This ring was missing in 11 of 12 cores collected from the largest SEGI tree sampled in GF. Douglass [3] made a special collection to confirm the existence of the 1580 ring, and it is the smallest ring in other SEGI chronologies as well [4]. Cores from LH were not deep enough, but those from two southern SESE locations (SPT, BB) also showed a strong low-growth marker for 1580. Multi-species dendroclimatic reconstructions for the region infer 1580 as a low water year [58], [61] and the 16^th^ century as a mega-drought [62]. One historical record from this time in California hints that low temperatures may also be involved. A written account of Chaplain Francis Fletcher from Sir Francis Drake’s voyage (published in 1628) described extremely cold conditions on the coast just north of San Francisco Bay in July 1579 [63]. More radial growth occurred in 1579 than 1580 (standard ring index of 0.793 vs. 0.519, respectively) at all SEGI sites, so effects of a severe 1579/1580 winter may have lingered into the 1580 growing season, further restricting radial growth.

### Dendroclimatic Relationships

As in other western North American forests, low moisture availability during dry summers appeared to constrain SESE radial growth [55], [64], [65]. The strength of this relationship was particularly noteworthy in the southernmost SESE location (Figure 11), where fires occurred in 1985 and 1999. Radial growth declined precipitously in the years immediately following these events, presumably as trees invested resources to rebuild fire-damaged crowns and restore leaf area. LH and other relatively dry SESE locations may thus be particularly useful for reconstruction of fire and drought histories. While SESE has generally been considered a complacent species [10] with low dendroclimatic potential, our results show that SESE ring widths do express meaningful climatic variation. Because our locations were all high-productivity forests with relatively ample access to soil water, sensitivities may be improved by sampling marginal locations and those closer to the inland and southern range limits [11], [46]. However, SESE sampling must be balanced by the need to maintain crossdatability, which may be more difficult in less productive forests.

Observed climate sensitivities and synchrony among tree-ring chronologies along the latitudinal gradient were congruent with northern, central, and southern portions of SESE’s geographic distribution [23]. SESE sub-regions showed different sensitivities to temperature: maximum spring temperatures correlated negatively with radial growth in central and southern locations, while minimum summer temperatures correlated positively with radial growth in rainforest locations. These relationships invoke a physiological interplay between maintenance costs, productivity, and optimum temperatures in tall forests [66]. For example, high spring temperatures may constrain radial growth by simultaneously increasing rates of maintenance respiration, elevating water stress, and decreasing gas exchange in relatively dry forests, whereas high summer temperatures may stimulate radial growth by promoting assimilation in cooler rainforests where water is not limiting. Such disparate sensitivities to temperature are particularly relevant for understanding the carbon sequestration capacity of SESE forests, because maximum and minimum temperatures have changed differentially over the last century with minimum temperature increasing at a faster rate, and overall temperatures in the region are projected to increase further during the 21^st^ century [67]–[70]. Accurately forecasting SESE responses to climate change will likely depend on consideration of such temperature-growth dynamics.

While summer fog is an important source of moisture in SESE forests [21], [22], [42], it can also obscure sunlight. Limited long-term historical and site-specific data in addition to complex climatological dynamics associated with the marine fog layer [71] have constrained investigation of the relationship between fog and radial growth [but see 72,73]. Reconstructed fog frequency derived from airport visibility records [42] provided a century-long time series along which to examine SESE radial growth, but these measurements were not specific to our locations and did not distinguish low clouds from within-canopy fog capable of delivering moisture in the form of crown drip or foliar uptake. All of our SESE locations occurred below 400 m elevation, and all but one location (BB) occurred far enough below this elevation that even the highest leaves of the tallest tree in the forest are <400 m elevation, so when fog is a problem at airports, nearby SESE forests are often merely being shaded. The negative relationship between SESE radial growth and summer airport fog frequency suggests that cloudiness reduced SESE radial growth by decreasing light availability for photosynthesis at the height of the growing season. Recent isotopic analyses of SESE tree rings from three northern locations support this interpretation. Middlewood cellulose was less enriched in ^13^C, which indicates less stomatal regulation of water loss and lower rates of CO_2_ assimilation, during years with higher summer fog (i.e., cloud base <200 m elevation) frequency, and this correlation strengthened and then stabilized with increasing cloud elevation, suggesting that the shading effects of cloudiness may influence radial growth more than canopy inundation by fog [20]. Latitudinal trends in correlations (Figure 8) imply that decreasing summer cloudiness (i.e., increasing light availability) has a significant positive effect on radial growth only in locations where water is least limiting. The fact that a central location (MW) exhibited the strongest negative correlation between radial growth and summer cloudiness makes sense, because this forest has perennial swamp-like conditions (personal observation) even though it receives only 60% of the average annual precipitation in northern rainforests (Figure 2). Clearly the relationship between fog and SESE growth warrants further study, especially in light of an inferred 33% reduction in summer airport fog frequency along the California coast since 1901 [42].

We confirmed findings of Hughes et al. [6], [18] that inter-annual variation in SEGI radial growth does not tightly match that of temperature or precipitation, but instead the smallest rings record extreme events such as severe droughts. Compared to SESE, significant correlations between radial growth and drought severity were relatively few in SEGI. However, PDSI does not account adequately for snowmelt and may be less reflective of actual soil moisture in SEGI forests, especially during the first half of the growing season [74]. June was a key climate month, as three sites (RMG, GF, FC) showed significant negative responses to temperature. Hot June temperatures likely cause SEGI, which has higher leaf water-use efficiency than SESE [75], to close stomata midday to avoid stress-induced embolism, thus curtailing photosynthesis during the longest days of the year. Positive correlations between radial growth and prior-October precipitation at two locations (WF, FC) combined with the lack of association between prior-October precipitation and snowfall at any location (single degree-of-freedom χ^2^, *P*>0.5) suggest that high rainfall in October extends the growing season, allowing SEGI to store more sugars overwinter for rapid trunk growth the following spring. Our analyses revealed only weak correlations between radial growth and snowfall with only one location (WF) showing significant responses to precipitation as snow and precipitation in January. We emphasize that these dendroclimatic relationships do not capture the potential lingering effects of snowpack, snowmelt, and the resultant elevated soil moisture, which demand research attention because snowmelt is occurring earlier in the year [76] and snowpack is projected to diminish in the Sierra Nevada even under low CO_2_ emission scenarios [77].

### Applications and Future Research

Crossdated SESE and SEGI tree-ring records have great potential to advance dendroclimatic, physiological, ecological, and archeological investigations of redwood forests. Our sampling technique of climbing and coring standing trees yielded sufficient replication to create baseline chronologies. Further replication will improve accuracy, and downed trees with ring-width records preserved in decay-resistant heartwood are now being utilized to extend sampling at multiple locations. For example, one very old remnant sample from HR has a ring count of 2,267 years with 742 years currently crossdated. Remnant wood can potentially be used to create multi-millennia tree-ring chronologies for climate reconstructions. In addition to the analyses presented here, our tree-ring data have been used to determine minimum tree ages [12] and crossdate branches to quantify branch growth dynamics [78]. Archeological applications of SESE chronologies are also emerging, including dating timber beams at the Presidio Officer’s Club in San Francisco, CA (Worthington, personal communication).

Another application of our SESE tree-ring chronologies involves creating and improving fire histories to guide forest management. Fire scars were observed on some cores, often at high trunk positions indicating crown fires. For example, a tree at JS had eight fire scars at different years on cores collected 45 and 78 m above the ground (Figure 1H). These observations have exciting potential to explore legacy effects of crown fires, which damage crowns and create decaying wood habitats important to a wide assortment of arboreal biota, including epiphytes and lungless salamanders [79], [80]. Extension of the northern SESE chronology back to the year 328 can enable accurate dating of fire scars previously observed in old samples [10], [19]. Crossdated SESE chronologies can be used to quantify precisely fire return intervals inferred from un-crossdated ring counts [81].

Our cross-dated samples are available for study of complementary tree-ring parameters. Additional measurements of latewood width and maximum latewood density may provide proxies for summer temperature or precipitation [82], [83]. Stable isotope composition of SESE wood cellulose can be assessed with inter-annual [20] and intra-annual [84] resolution, and crossdated samples provide dated material essential for such analyses. For this study, we detrended tree-ring chronologies to focus on inter-annual variation. We are also exploring lower-frequency variation in ring widths to quantify long-term trends in aboveground wood production and carbon sequestration, and we continue to monitor SESE and SEGI as part of ongoing research in a growing network of permanent plots.

## Supporting Information

Figure S1 **Average monthly precipitation and maximum and minimum temperature for northernmost and southernmost redwood locations.** For each location, values are 114-year averages (1895–2008) at 800 m resolution using PRISM data [36].
(TIF)

Figure S2 **Low-growth marker years in tree-ring chronologies from 14 locations for two species (1500–2008).** Marker years were ten smallest ring widths per century at each location after removing years with three or fewer locations. LH chronology ended 1653.
(TIF)

Table S1 **Tree-ring chronology and replication data for eight** ***Sequoia sempervirens*** **and six** ***Sequoiadendron giganteum*** **locations.** Standard and residual chronologies using *high crossdating confidence* series >50 years detrended with 32-year spline.
(XLSX)
