# Supplementary figures and images for: Millennium-Scale Crossdating and Inter-Annual Climate Sensitivities of Standing California Redwoods
Source: PLoS One. 2014 Jul 16;9(7):e102545. doi: 10.1371/journal.pone.0102545 (PMC4102271; doi:10.1371/journal.pone.0102545)

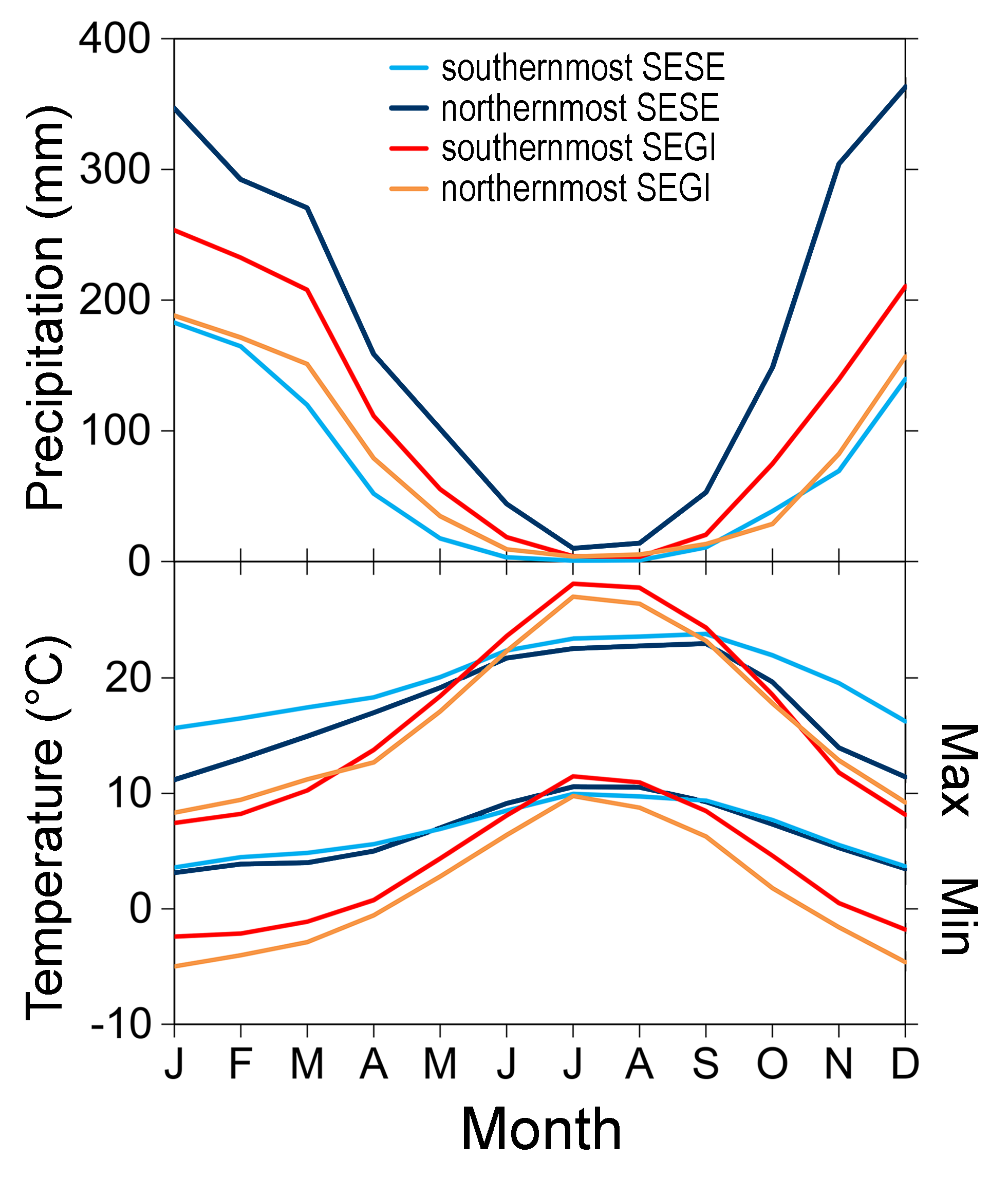

Supplement: Figure S1 — Average monthly precipitation and maximum and minimum temperature for northernmost and southernmost redwood locations. For each location, values are 114-year averages (1895–2008) at 800 m resolution using PRISM data [36]. (TIF) [file pone.0102545.s001.tif]

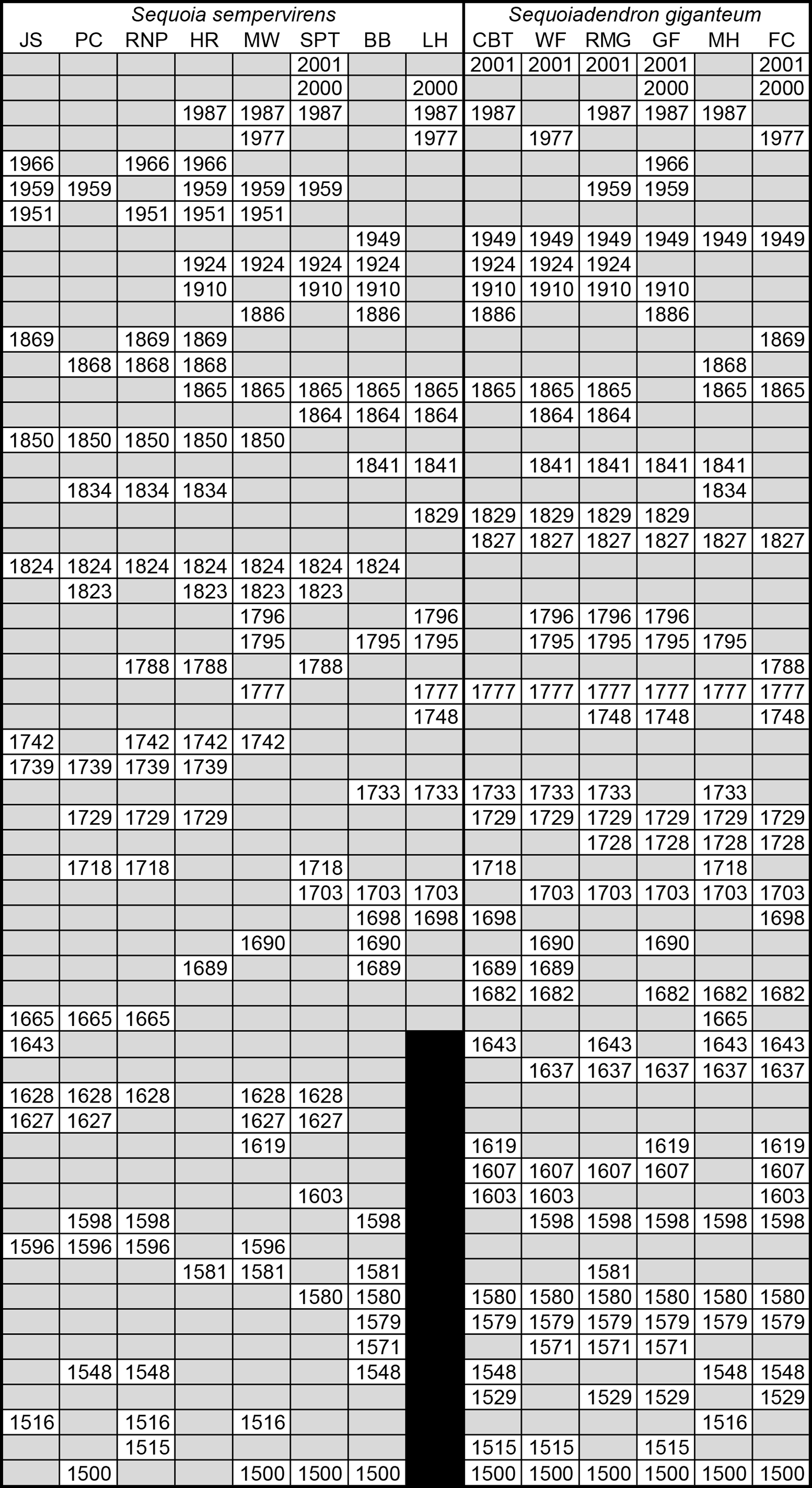

Supplement: Figure S2 — Low-growth marker years in tree-ring chronologies from 14 locations for two species (1500–2008). Marker years were ten smallest ring widths per century at each location after removing years with three or fewer locations. LH chronology ended 1653. (TIF) [file pone.0102545.s002.tif]
